# Apparent Deterioration Law and Shear Failure Mode of Rock–Mortar Interface Based on Topography-Sensing Technology

**DOI:** 10.3390/ma16020763

**Published:** 2023-01-12

**Authors:** Linglin Xie, Wenyu Tang, Hang Lin, Fan Lei, Yifan Chen, Yixian Wang, Yanlin Zhao

**Affiliations:** 1 Key Laboratory of Natural Resources Monitoring and Supervision in Southern Hilly Region, Ministry of Natural Resources, Changsha 430071, China; 2 The Second Surveying and Mapping Institude of Hunan Province, Changsha 430071, China; 3 School of Resources and Safety Engineering, Central South University, Changsha 410083, China; 4 China School of Civil Engineering, Hefei University of Technology, Hefei 230009, China; 5 School of Energy and Safety Engineering, Hunan University of Science and Technology, Xiangtan 411201, China

**Keywords:** topography-sensing technology, rock–mortar interface, cyclic shear loading

## Abstract

As an advanced spatial technology, topography-sensing technology is comprehensive, macroscopic, and intuitive. It shows unique advantages for rock structure interpretation and has important guiding significance for the research of the shear performances of rock–mortar interface under cyclic load in rock mass engineering. In this paper, cyclic shearing tests combined with the shear surface topography-sensing technology are employed to investigate the evolution characteristics of the interface morphology and the strength deterioration of the rock–mortar interface. Primarily, mortar and three types of rocks are used to prepare different rock–mortar interfaces, which are then applied to cyclic shear loading under two constant normal stresses. Subsequently, the shear strength degradation and dilatancy characteristics of rock–mortar interfaces with varying shear times are discussed. In addition, on the basis of the non-contact three-dimensional topography-sensing technology, the apparent three-dimensional point–cloud coordinate information of rock–mortar interface before and after each shear loading is obtained, and the apparent three-dimensional topography parameters of rock–mortar interface are calculated, according to which the influences of normal stress and lithology on the topography of interface subjected to cyclic shearing loading are analyzed.

## 1. Introduction

**Figure 1:**
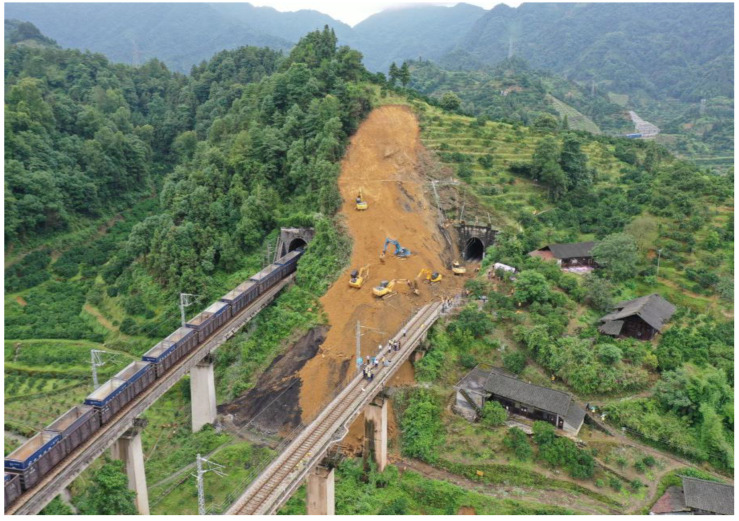
Landslide in engineering.

Engineering rock mass contains a large number of joints and fractures with different scales, the occurrence of these defects greatly affects the integrity of rock mass and reduces the rock strength [1,2,3]. The properties of joint interface determine the mechanical responses of rock mass to varying degrees and then affect the safety and stability [4,5]. Multitudinous engineering instability disasters of rock mass are related to shear slip of joint interfaces [6,7,8], as shown in Figure 1. 

Generally, the shear strength of rock interface is closely related to its apparent morphology [9]. Barton and Choubey [10] first proposed using 10 standard profile lines to characterize the morphological characteristics of joints. By introducing joint roughness coefficient *JRC*, the well-known *JCS-JRC* evaluation model of interface shear strength was established. Zhao [11,12] built the JRC-JMC shear strength model considering the block anastomosiation during shearing. Maksimović [13] used the rough angle Δ*φ* and the median angular pressure value *P_N_* to represent the peak dilatation angle and established the shear strength model. Through studying the fractal dimension of interface, Kulatilake et al. [14] proposed a shear strength model that could characterize the interface anisotropy. Homand et al. [15] defined multiple parameters to describe the characteristics of the three-dimensional interface, such as fluctuation, inclination, and curvature and deduced the shear strength model. Singh and Basu [16] collected 11 reasonable criteria for peak shear strength and found that the Tang model [17] and Yang model [18] based on Grasselli morphological parameters were superior to other models in form and prediction accuracy. In addition, the exploration of interface morphology cannot be separated from the corresponding equipment. The perspective of topography-sensing technology is macro and comprehensive [19], which is convenient for interface apparent topography investigation and model construction of rock mass, wherein the three-dimensional non-contact laser topography sensor is the most widely used surface shape measurement equipment currently [20]. The parameter estimation is very intelligent, and it is easy to obtain the data of fractal geometric parameters, statistical parameters, height parameters, and texture characteristics, which is conducive to the subsequent research on the mechanism of interface topography degradation. Xia and Sun [21,22] designed the RSP-1 type intelligent rock surface topography analyzer and the TJXW-3D type three-dimensional rock surface topography sensor successively to achieve better measurement of surface topography of rock interface. Cao and Fan [23] used Talysurf CLI 2000, a high-precision three-dimensional surface topography sensor, to scan the interface surface for realizing the three-dimensional visualization of interface. Grasselli et al. [24,25,26] proposed employing the maximum potential contact area ratio, maximum apparent angle, and roughness parameters to quantitatively describe the interface topography characteristics through high-precision three-dimensional scanning and sensing quantification method of interface topography. Gui et al. [27,28] studied the shear mechanical behavior and the deterioration characteristics of interface topography in different shear displacements on the basis of three-dimensional topography-sensing technology. Although these have greatly enriched the understanding of shear mechanical properties of rock interfaces, most are concentrated on the single shear performance of the interface.

In fact, cyclic load is a more common occurrence than static load in the destruction of rock and soil structures [29,30,31]. Under the action of cyclic shear load, the joint interface will continuously slip and close, and the rough micro-convex on the interface will suffer periodic wear and passivation, leading to the deterioration of shear strength parameters [32,33]. Then, the rock mass is staggered and slipped along the interface, and eventually results in failure [34,35,36]. Therefore, studying the mechanism of cumulative damage and shear strength deterioration of rock interface under cyclic load is one of the key scientific problems to accurately evaluate the dynamic stability of rock mass [37,38]. Lee et al. [39] conducted cyclic shear tests on saw-split joint interfaces and studied the shear mechanical behavior. Jafari et al. [40] conducted cyclic shear tests on artificial joint interfaces to analyze the influence of shear cycle times and normal stress. Mirzaghorbanali et al. [41,42], and Niktabar et al. [43] carried out a series of cyclic shear tests under constant normal stiffness boundary conditions to explore the influence of initial normal stress and shear rate. Niktabar and Rao [43] developed a new type of large servo-controlled direct shear testing machine, on which the cyclic shear tests of different shear load frequency, amplitude, and normal stiffness under constant normal stiffness and constant normal stress boundary conditions can all be realized. Liu and Dai [44] revealed the influences of loading frequency, maximum stress, and amplitude on the mechanical properties of rock interface through laboratory tests. Fan and Jiang [45] found that the fatigue life of samples decreased significantly under the action of discontinuous cyclic compression. Zhang and Dong [46] proposed the formula for calculating the basic internal friction angle and dilatancy angle of rock interface under cyclic shear loading and then established the calculation method for the peak shear strength of interface considering the deterioration of both angles. Wu and Jiang [47] ponited out that the influence of cyclic loading on the shear capacity of bolt is much greater than that on the rock interface. Kou and Liu [48] stated that wear failure occurred at a low number of cycles, while fatigue crack initiation and propagation occurred at a high number of cycles. Dang and Konietzky [49] proposed a mathematical formula to predict the shear strength of rock interface under cyclic changes of shear velocity and normal load. It is not difficult to conclude that the above focuses mainly on the influence of loading conditions on mechanical properties, and there are few reports on the analysis of the morphology degradation of interface under cyclic loading. In addition, the test samples are homogenous rock interfaces.

**Figure 2:**
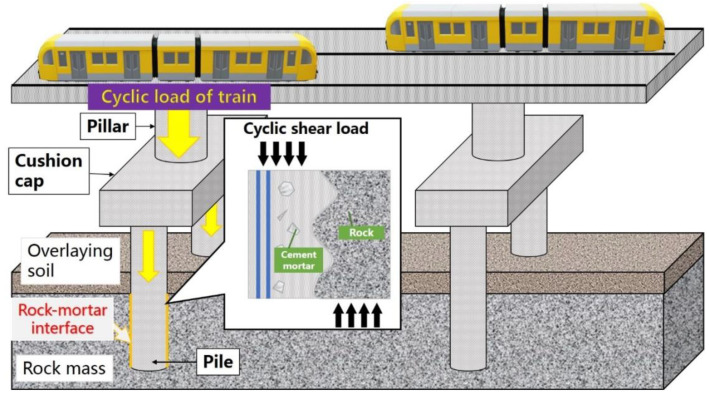
Interface between rock-socketed pile and bedrock in high-speed railway.

As a common construction and reinforcement material, cement mortar material is widely used in rock mass reinforcement, tunnel excavation, highway and bridge construction, and other rock mass engineering due to the excellent performance [50,51,52], generating numerous rock–mortar interface structure [53,54], which also bring huge workload and difficulty to the safety management and disaster prevention in the construction and operation of basic transportation facilities [55,56,57]. Considering bridge operation as an example, as shown in Figure 2, rock-socketed piles and bedrock are cemented to provide support for piers and beams on the bridge, while the stability of rock–mortar cementation interface is the basis of safe and stable train operation. On the other hand, trains generate loads when passing over the bridge. The load acts on the interface between rock-socketed pile and bedrock through the bridge piers, forming cyclic shear stress, which poses a great threat to the safety and stability of the bridge. Therefore, it is of great practical significance to study the shear mechanical properties of rock–mortar interface under cyclic shear loading.

In this paper, samples with different rock–mortar interfaces are prepared to carry out cyclic direct shear tests under multi-normal stress conditions. The influences of rock types, normal loads, and shear times on the apparent morphology evolution of the interface are studied, respectively. Meanwhile, the digital three-dimensional topography-sensing technology is used to collect the interface appearance before and after shearing, which is characterized by multiple three-dimensional topography parameters. Subsequently, the evolution of the parameters representing the interface volatility, dispersion, anisotropy, texture characteristics, interface complexity, peak point, steepness, and effective shear body height of mortar and rock dual medium interface are respectively analyzed. The research results have important practical significance for rock engineering safety construction, resource saving, and disaster warning.

## 2. Presentation of Test Strategy

### 2.1. Test Apparatus

(1) Topography-sensing test of rock–mortar interface

**Figure 3:**
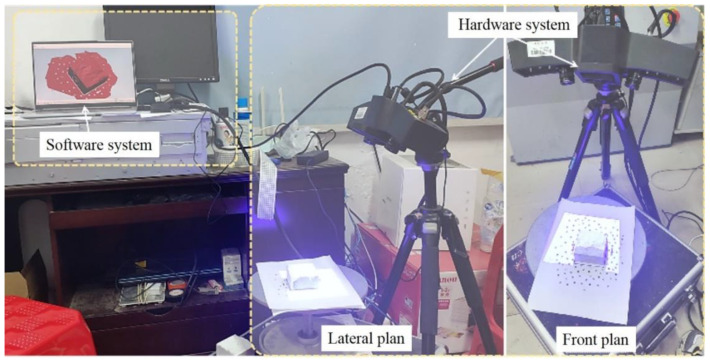
The components of interface topography-sensing system.

**Figure 4:**
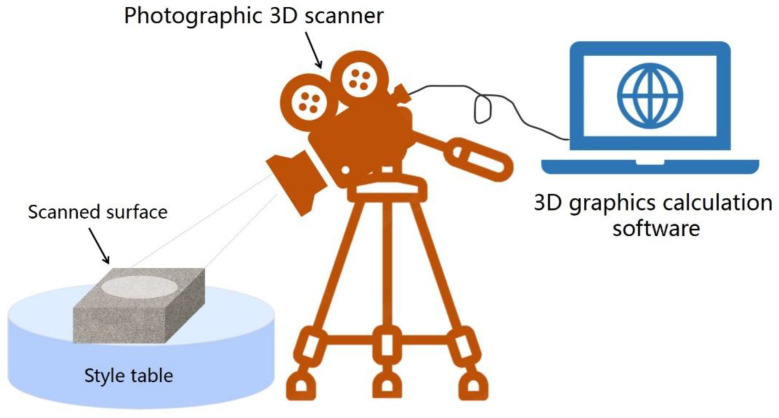
The calibration of topography-sensing system.

The interface topography-sensing system used in this test is HL-3DX photo-type three-dimensional scanning system produced by Hualang three-dimensional Company. Its system is composed of a hardware system and software system (see Figure 3). The former is responsible for taking photos and sampling, and the latter serves for stitching and integrating. Before the interface topography sensing and modeling, it is necessary to adopt the latest international coding calibration technology to calibrate the system, as shown in Figure 4. This is the core grasp and application of three-dimensional photogrammetry technology, which improves the original three-dimensional data scanning accuracy. By using the granite material calibration version, excellent thermal stability can be obtained, and the scanning measurement accuracy will be greatly improved. In addition, Blu-ray scanning device and multi-frequency phase-shift grating technology are adopted to enhance anti-jamming ability and improve data quality. In this test, the spacing between scan data points is set as 0.01 mm.

(2) Cyclic direct shear tests on rock–mortar interface

**Figure 5:**
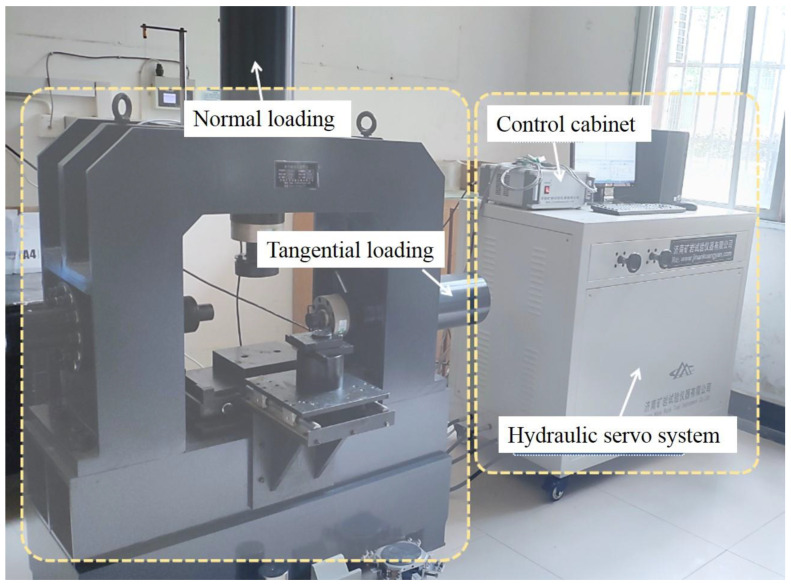
ZW100 multifunctional rock direct shear instrument.

ZW100 multifunctional rock direct shear instrument (see Figure 5) is applicable mainly to the direct shear tests of rock structural surface (such as interface, bedding, schistosity, fracture surface, etc.), rock, and the bonding interface of concrete or mortar and rock. It is automatically controlled by the computer, that is, speed, force, displacement, and other parameters are displayed in the computer control, all of which can be automatically analyzed and calculated in the database. The test curves are recorded, displayed, and saved in real time with high-speed sampling during the test process, wherein the displacement measurement is performed diagonally with four displacement gauges. Beyond that, the instrument is rated to 500 kN in normal direction and 500 kN in tangential direction. Normal space is less than 450 mm, and transverse space is less than 200 mm. Two loading modes of force control and displacement control are optional for tangential loading. Specifically, the loading speed of force control is in the range of 0.01–20 kN/s, the loading speed of displacement control is in the range of 0.01–60 mm/min, the load accuracy is 0.01 kN, and the loading mode is electro–hydraulic stress type. Considering the sample condition and the operation convenience, constant force control is selected for normal loading, while the combination of constant force control and constant displacement control is used in tangential loading finally. The specific loading process is showing as follows:

- Before loading, the normal and tangential loading devices are moved close to the sample quickly and stopped at approximately 5 mm away from the sample.
- Normal loading is controlled by constant force, and the loading speed is 0.1 kN/s until the target value is reached. Tangential loading begins after the normal force remains stable for 10 s.
- Tangential loading is achieved by constant force and constant displacement: Firstly, tangential force control is adopted at 0.1 kN/s until the tangential force reaches 2 kN. Then, the tangential displacement control is manually started to load at a constant speed of 0.01 mm/s, and it ends when sample failure occurs.

### 2.2. Rock–Mortar Interface Preparation

**Figure 6:**
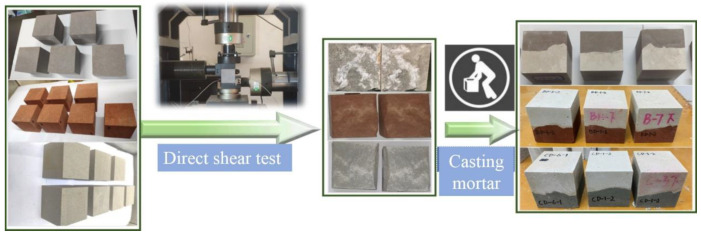
Rock–mortar interface preparation flow.

As shown in Figure 6, the rock–mortar interface samples formed by three types of rocks and mortar are prepared [58,59]. The three rocks are, respectively, malmstone, red sandstone, and blue sandstone, which are denoted as A, B, and C. Firstly, the natural irregular interfaces are obtained by direct shear tests on the complete rock samples (cubic rock with the length of 70 mm), and the basic mechanical parameters of the three rocks are measured as shown in Table 1. These irregular interfaces are then scanned as the intial three-dimensional morphology of interfaces.

The rock blocks with clear failure surface, complete structure, and different roughness are selected and placed in the 70 mm × 70 mm × 70 mm mold. Then, cement mortar is cast on the rough surface side of rock blocks. Note that mortar is made of #425 cement, fine sand, and water in the ratio of 2:1:0.6. The samples are placed in the standard curing box for 28 days. In addition, to reduce the influence of sample surface flatness on the test accuracy, each surface is polished to ensure that the loading can be evenly applied to the samples.

### 2.3. Test Program

The test loading scheme is presented in Table 2. The sample number AD-1 refers to the interface sample prepared by malmstone and mortar. After each shear test, the shear surface is scanned to obtain the interface topography parameters. A total of 24 direct shear tests and 32 interface topography scans are carried out.

## 3. Strength Deterioration and Dilatancy Characteristics of Rock–Mortar Interface

### 3.1. Deterioration of Interface Strength under Cyclic Shearing

**Figure 7:**
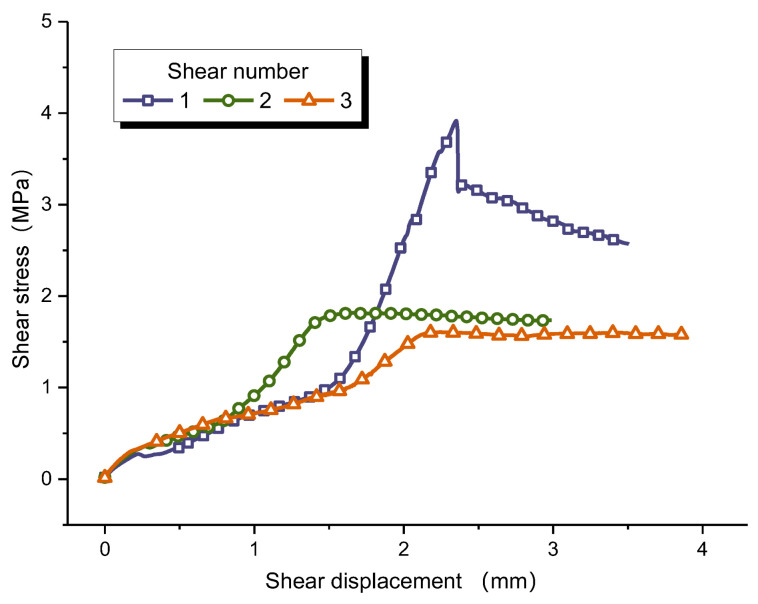
The shear stress vs shear displacement curves of sample CD-1 in each shearing.

After cyclic direct shear tests, both the shear strength and shear-stress–shear-displacement curves of the interface greatly changed [8,60]. Since the curve variation trend of all interfaces is roughly similar, only representing curves are listed to save space. Figure 7 shows the relationship between shear stress and shear displacement of sample CD-1. During the first shearing, the shear-stress–shear-displacement curve drops rapidly after the peak, and the failure mode of the interface is brittle failure. However, the shear stress decreases slowly after the peak in the second shearing, and the failure mode turns out to be slip failure. Next shearing, the shear stress remains stable after yielding. This indicates that the failure mode of rock–mortar interface changes from brittle fracture to friction sliding during cyclic shearing, the shear strength decreases continuously, but the shear stress drop in the post-peak gradually decreases, which means gentler energy release. Therefore, in the engineering, although the bearing capacity of the structural plane subjected to cyclic shear decreases, it can effectively avoid sudden disasters when the structural instability occurs, and it provide more evacuation time for personnel and equipment.

Table 3 summarizes the shear strength of each rock–mortar interface after each shearing. Obviously, it decreases with the increase of shearing times. Upon comparing, it is seen that the shear strength of rock–mortar interface prepared by malmstone is highest, while the reduction of shear strength by cyclic shearing is relatively low.

**Figure 8:**
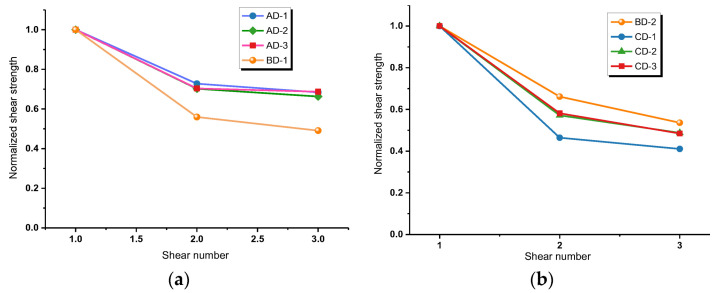
Normalized relationship between shear strength and shear times. (**a**) Normal stress = 4 MPa; (**b**) Normal stress = 2 MPa.

For the convenience of comparative analysis, the shear strength in Table 3 is normalized to obtain the relationship between the normalized shear strength and shear times, as shown in Figure 8. It can be seen that the interface shear strengths are reduced by 70%, 50%, and 45% after the second shearing for blue sandstone, red sandstone, and malmstone, respectively. The results prove that in terms of cyclic shear properties of rock–mortar interface, malmstone is superior to red sandstone, and both of them are better than blue sandstone. In addition, through comparing the shear stress variation of BD interfaces, it can be concluded that the strength reduction effect is more prominent for the sample with larger normal stress. The rapid reduction of structural bearing capacity under cyclic shearing is one of the decisive factors for the stability of similar projects, while appropriate reduction of normal stress can lower the decay rate of bearing capacity in a certain range.

### 3.2. Dilatancy Characteristics of Interface under Cyclic Shearing

**Figure 9:**
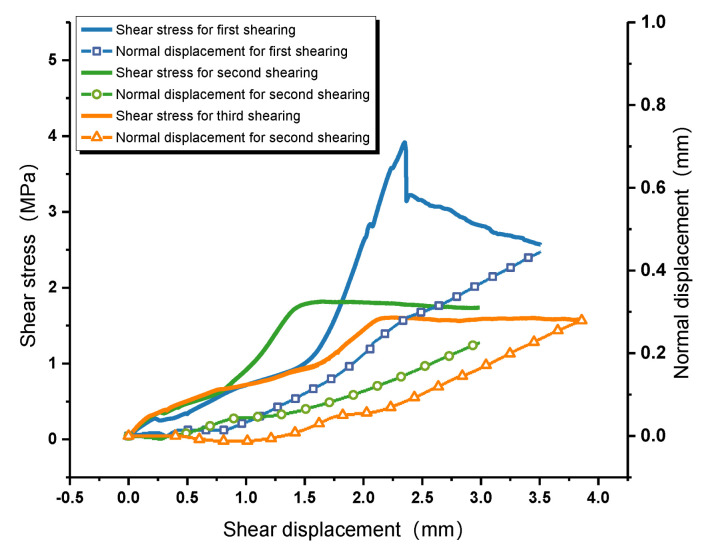
The relationship between normal displacement and shear displacement.

**Figure 10:**
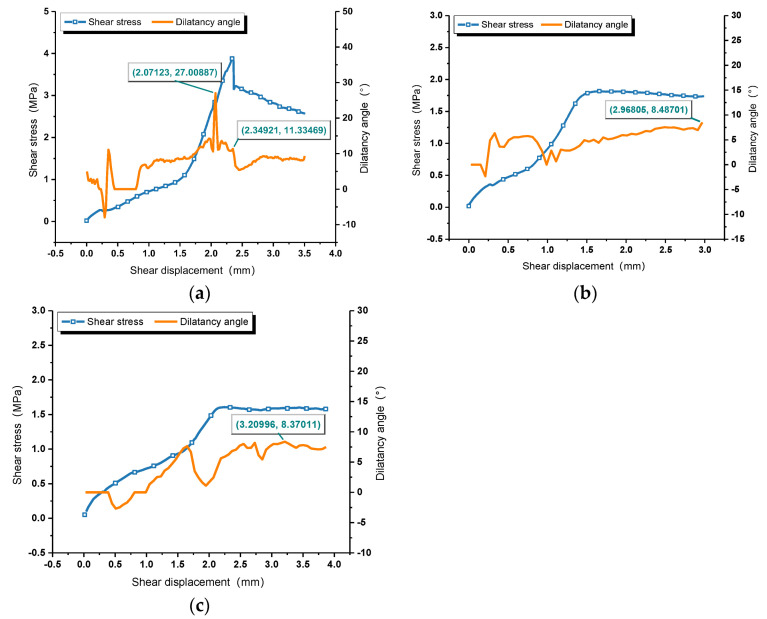
The relationship between shear displacement, dilatancy angle, and shear stress. (**a**) First shearing; (**b**) Second shearing; (**c**) Third shearing.

The relationship between normal displacement and shear displacement is described in Figure 9. Due to the existence of irregular rough interfaces between rock and mortar, the normal displacement increases with the increase of shear displacement, while it fluctuates before the peak shear stress [61,62]. After that, a linear relationship is shown. Each shearing will wear and cut off the microscopic bulges on the interface, which means that the longer the interface shear times, the smaller the normal displacement and the smaller the dilatancy effect. As defined, the real-time dilatancy angle is the dip angle between the tangent line and the horizontal plane at each point of the dilatancy curve. According to a definition by Barton, the real-time dilatancy angle at the peak shear displacement is the the peak dilatancy angle *d_n_*. Figure 10 shows the relationship between dilation angle, shear stress, and shear displacement of each shearing. The peak dilatation angle after first shearing is 27°, while the dilatancy angle corresponding to the peak shear stress is 11.33°, which shows a great difference. In the process of practical interface shearing, the maximum dilatancy effect is earlier than shear failure due to the different stiffnesses of mortar and rock. Thus, it is more appropriate to regard the the peak value of the real-time dilatancy angle curve as the peak dilatancy angle *d_n_*. Considering that there is no obvious peak shear stress in the shear-stress–shear-displacement curves of the second and third shearing, the maximum dilatancy angles are employed, which are 8.487° and 8.37°, respectively. With the increase of shear numbers, the values of *d_n_* decreases at a decreasing rate.

**Figure 11:**
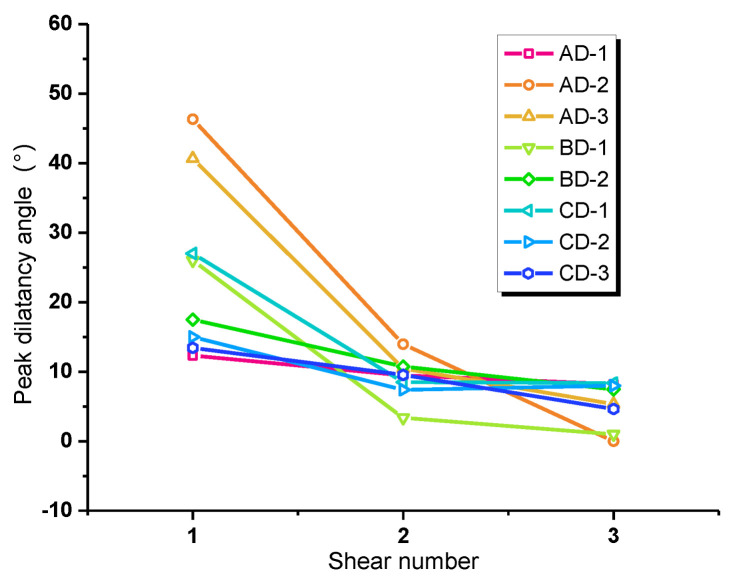
Relationship between shear times and peak dilatancy angle *d_n_*.

As can be observed in Figure 11, *d_n_* of the first shearing ranges between 15° and 50°, *d_n_* of the second shear is distributed in the intervel from 5° to 15°, and *d_n_* of the third shear changes from 0° to 10°. With the increase of shear times, the peak dilatancy angle decreases, and the interface with larger normal stress has a greater reduction of peak dilatancy angle. Meanwhile, the differences between the peak dilatancy angles of each interface are getting smaller, indicating that its sensitivity to the normal stress, the interface material, and the roughness is decreasing. However, by comparing the peak dilatancy angle of the first shearing, it can be found that the dilatancy angle corresponding to malmstone is generally larger, followed by red sandstone and blue sandstone.

## 4. Apparent Evolution Characteristics of Rock–Mortar Interface

### 4.1. Introduction of Three-Dimensional Topography Parameters

**Figure 12:**
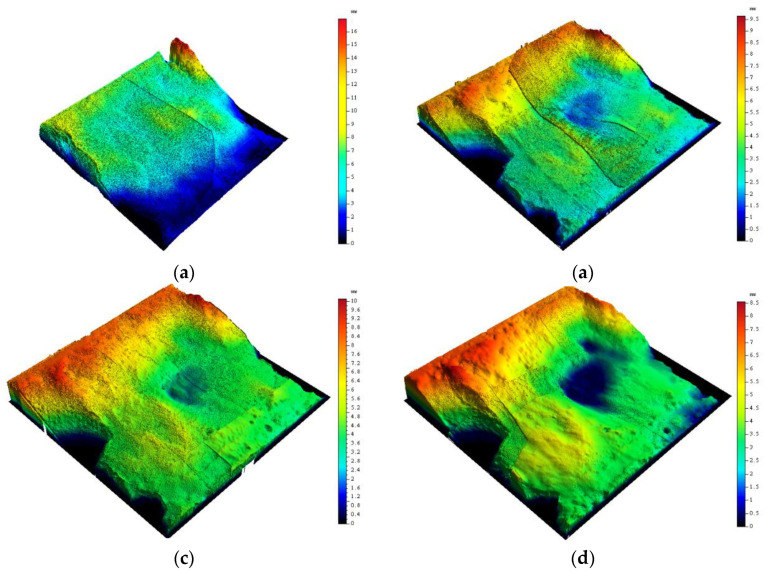
The three-dimensional scanning of CD-3 interface for each shearing. (**a**) Before shearing; (**a**) Before shearing; (**c**) Second shearing; (**d**) Third shearing.

Topography-sensing technology is widely used in engineering [63,64,65]. To study the morphology changes of the rock–mortar interface after cyclic shearing, the initial morphologic scan is carried out for each interface after each shearing. The scanning accuracy is 0.01 mm, and four scanning tests are implemented for each interface. The three-dimensional scanning of CD-3 interface is presented in Figure 12.

After scanning the failure interface, the post-processing software is used to calculate the characteristic parameters of the interface topography. The specific parameters are as follows:

(1) Description of height characteristic parameters of surface topography.
  a. Maximum interface height *S_p_*: The vertical distance between the lowest point and the highest point of the interface;
  b. Interface height root mean square *S_q_*: The square root of the mean distance from each point on the measured surface to the datum. The expression is
    (1) $$S_{q}=\sqrt{\frac{1}{A}\iint_{A}z^{2}(x,y)dxdy}$$
  c. Arithmetic mean deviation *Sa*: The arithmetic mean of the distance from each point on the measured surface to the datum in the sampling area, which can be calculated by
    (2) $$S_{a}=\frac{1}{A}\int_{A}\left|z(x,y)\right|dxdy$$
  d. Skewness coefficient of surface height distribution function *S_sk_*: The ratio of the cubic moment of the height distribution density function to the third power of the root mean square of height. It describes the symmetry of the surface topography and can be expressed by Equation (3).
    (3) $$S_{sk}=\frac{1}{S_{q}^{3}}\left(\frac{1}{A}\iint_{A}z^{3}(x,y)dxdy\right)$$
  e. The peak coefficient of the surface height distribution function *S_ku_*: Description of the steepness of the interface surface topography. The calculation formula is
    (4) $$S_{ku}=\frac{1}{S_{q}^{4}}\left(\frac{1}{A}\iint_{A}z^{4}(x,y)dxdy\right)$$

In the above formula, *z*(*x*,*y*) is the surface height distribution function, and *A* is the sampling area of the structural plane.

(2) Surface morphology texture feature parameters
  a. Texture topography ratio *S_tr_*: Characterizing the degree of interface anisotropy and anisotropy. Its values range from 0 to 1. The larger the value, the greater the degree of isotropy.
  b. Texture direction angle *S_td_*: Describing the main tilt direction of the surface texture. The value is the angle between the main tilt direction and the tilt direction of the least square surface.
(3) Surface peak morphology characteristics
  a. Peak point density *S_pd_*: Number of peak points per unit area, which can characterize the complexity of the surface.
  b. Arithmetic mean curvature at peak point *S_pc_*: Arithmetic mean of curvature of all peaks, which reflects the surface peak’s sharpness or roundness.
(4) Surface topography mixed parameters
  a. Fractal dimension *D_s_*: The box-counting method is used to calculate fractal dimension of the surface, and the slope of the regression line is the fractal dimension, which can describe the complexity of the interface.
  b. Root mean square of slope of interface *S_dq_*: Characterizing the tilt degree of the interface to a certain extent, which can be obtained by
    (5) $$S_{dq}=\sqrt{\frac{1}{A}\iint_{A}{\left(\frac{\partial z(x,y)}{\partial x}\right)}^{2}+{\left(\frac{\partial z(x,y)}{\partial y}\right)}^{2}dxdy}$$
  c. Interface area ratio *S_dr_*: Used to represent the complexity of the interface, the expression of which is

(6) $$S_{dr}=\frac{1}{A}\left(\iint_{A}\left(\sqrt{1+{\left(\frac{\partial z(x,y)}{\partial x}\right)}^{2}+{\left(\frac{\partial z(x,y)}{\partial y}\right)}^{2}}-1\right)dxdy\right)$$

### 4.2. Morphology Parameter Evolution of Rock–Mortar Interface Subjected to Cyclic Shearing

**Figure 13:**
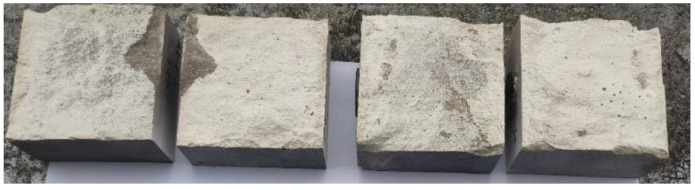
Failure surfaces of AD-1 and AD-2 after the first shearing.

**Figure 14:**
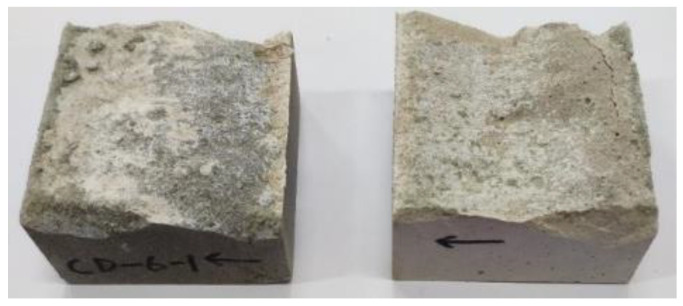
Failure surfaces of CD-3 after the first shearing.

Table 4 summarizes the height topography parameters of each interface before and after cyclic shearing. Generally, the maximum height *S_p_* decreases with the increase of shear times, as in the four groups of interfaces with 4 MPa normal stress (AD-1, AD-2, AD-3 and BD-1); however, *S_p_* corresponding to first shearing is greater than that before the test and then shows a decreasing trend with the increase of shear times. The reason is that mortar and rock bond more closely under greater normal stress, and the two materials share the shear resistance in the shearing process. During the first shearing, the two materials are not completely separated, and part of the mortar remained bonded to the rock surface. In other words, a new failure surface occurred (see Figure 13), producing greater roughness. In the second and third shearing, the micro-convex is cut off, causing the decrease of *S_p_*. For the other interfaces (BD-2, CD-1, CD-2, and CD-3), the bonding between mortar and rock is relatively weak [66]. Under the action of shear stress, the interface is worn and damaged after separation, as shown in Figure 14.

The arithmetic mean difference of surface height *S_a_* decreases with the increase of shear times under 2 MPa normal stress, but it irregularly fluctuates under 4 MPa normal stress, which can be attributed to the great changes of interface morphology. Compared with *S_a_*, the interface height deviation root mean square *S_q_* is not only related to the relative foundation surface but also more sensitive to the interface points, which can better reflect the volatility and dispersion of interface. The variation pattern of *S_q_* is similar to that of *S_p_*, that is, with the increase of shear times, *S_q_* shows a decreasing trend. This indicates that the dispersion of the interface height distribution is weakened due to the damage of micro-convex. The higher micro-convex are worn or cut off, while the lower micro-convex show little changes.

If the skewness coefficient of the surface height distribution function *S_sk_* is less than 0, there are more thin and deep valleys on the surface, and vice versa. While the peak coefficient of the surface height distribution function is greater than 3, the height distribution is more concentrated, whereas the height distribution is more discrete. There is no obvious regularity in the changes of *S_sk_* in Table 4 because most of *S_sk_* tends to 0, which means the peaks and the troughs of interface tend to be symmetrical. However, the crest is constantly worn in the tests, while the number of troughs is almost constant, resulting in more discrete height distribution of interface. In other words, *S_ku_* shows a downward trend. Considering that there is a fourth power term in the *S_ku_* calculation formula, it is not sensitive to the distinction between peaks and troughs. Therefore, the validity of this parameter in describing the uneven surface remains to be further investigated. It is suggested to avoid employing the above two parameters in the topography description of interface subjected to cyclic shearing.

If the texture aspect ratio *S_tr_* approaches 1, the interface topography has a high degree of isotropy. While *S_tr_* is close to 0, it is highly anisotropic. In Table 5, the values of *S_tr_* before the tests are all less than 0.5, indicating that the anisotropy of texture morphology at the interface is obvious in the process of cyclic shearing. Since the irregular interface before tests is obtained by the intact rock shear tests, the corresponding *S_tr_* are also less than 0.5. In addition, *S_tr_* increases with the increase of shear timess, which means that the differences on the interface morphology in different directions gradually decrease. In cyclic shearing, due to the gradual wear of the micro-convex, the elevation difference of each point is gradually loaded. In addition, due to extrusion, the convex and concave parts gradually merge to form a micro-surface with fluctuation. The original tiny folds on the interface are also smoothed out or formed into small curved surfaces with low undulation angles. All these decrease the anisotropy of the interface, causing the increase of *S_tr_*. However, when the normal stress is 4 MPa, *S_tr_* of AD-1 and AD-2 interface after the first shearing is smaller than that before the tests, indicating that the first shearing raises the interface anisotropy and produces more complex interfaces. 

As can be seen in Table 5, the texture direction angle *S_td_* decreases with the increase of shear times, and the decreasing amplitude is related to the normal stress. The protruding parts on the interface are gradually worn down during the cyclic shearing process, resulting in gradual decreases in the height, which is the main cause of *S_td_* reduction. Under low normal stress, the decreasing amplitude is relatively small. This is due to the weak extrusion between rock and mortar under 2 MPa normal stress. In the shearing process, the failues that occurred most often are slips along the direction of texture angle and wear damage to the small sharp convex body on the texture body, while the wear on the texture body is very weak. As a result, *S_td_* is less sensitive to changes in the texture features of the interface.

The variation of peak density *S_pd_* can describe the complexity of the surface in the mesoscopic dimension. It ranges from 0 to 1 and decreases with the increase of shear times shown in Table 5. The decrease of *S_pd_* is attributed mainly to the fact that high peaks on the measured surface are smoothed out in the process of cyclic shearing, resulting in a flatter surface. Especially in the case of large normal stress, higher peak points are mostly cut off or worn down during the first and second shearing, and there is less change of *S_pd_* after the third shearing. 

The variation of *S_pc_* is similar to that of *S_pd_*. The reduction of *S_pc_* indicates that in the process of cyclic shearing, shear failure or friction failure occurs to the steep and sharp convex on the interface under the normal and shear stress, lowering the height difference between the peak point and surrounding area. Moreover, the steepness of local areas decreases, forming a smoother and more rounded peak point. 

**Figure 15:**
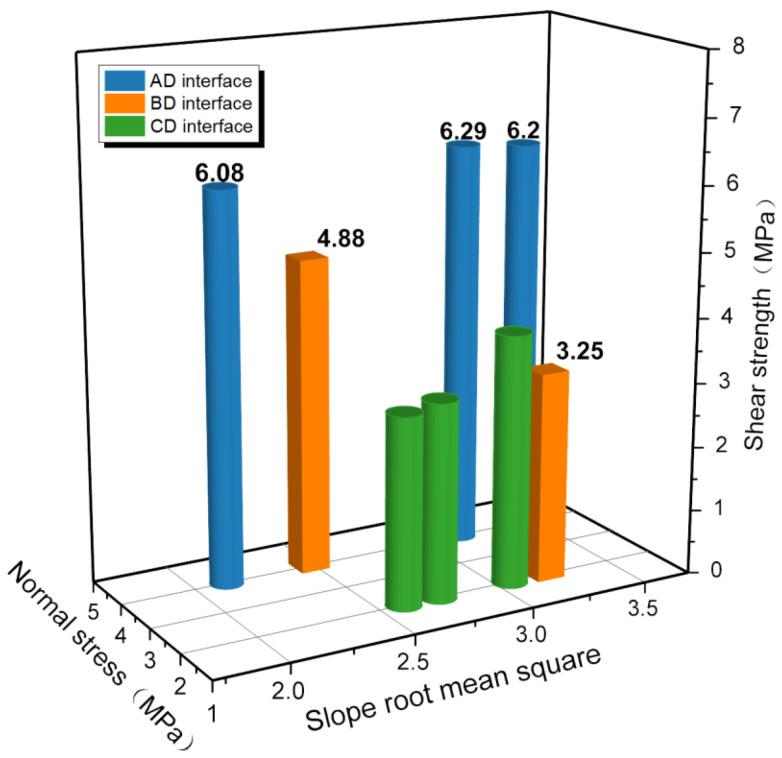
Relation between slope root mean square and shear strength.

Slope root mean square is an important parameter to describe interface topography. Table 6 presents a decreasing trend of slope root mean square in cyclic shearing, which reveals that the surface morphology of interface is becoming gentle. The relationship between the interface shear strength and the slope root mean square is plotted in Figure 15. The blue bar chart is the shear strength of AD interface under 4 MPa normal stress, the green bar chart shows the shear strength of CD interface under 2 MPa normal stress, and the orange bar chart shows the shear strength of BD interface under 2 MPa and 4 MPa normal stress. As the slope root mean square changes, the shear strength of AD interface shows little change under the normal stress of 4 MPa, while the shear strength of CD interface increases by 31.6% under the condition of 2 MPa normal stress. This proves that the interface morphology has a greater influence than normal stress on the shear strength under the condition of small normal stress. In shallow strata with low in situ stress, increasing the roughness of the interface will greatly improve the reinforcement effect of anchorage and grouting. Since the interface roughness has little influence on the shear strength of the interface under 4 MPa normal stress, it can be approximated that the shear strength of BD interface with slope root mean square of 3.3 is approximately 4.88 MPa. That is, when the normal stress increases by 100%, the shear strength is enhanced by approximately 50.2%.

When the expansion area ratio of the surface *S_dr_* approaches 0, it indicates that the tested surface is almost completely flat. In Table 6, the value of *S_dr_* decreases with the increase of shear times. Under the action of cyclic shearing, the uneven parts of the interface are constantly worn and cut off, and the wrinkles and the expansion area are reduced. Under the large normal stress, *S_dr_* decreases faster. This is because the normal stress promotes the contact between rock and mortar, which makes the shearing wear effect more obvious.

Fractal geometry is proposed by French mathematician Benoft Mandelbrot to describe and analyze the scale invariance phenomenon and has gradually become an important tool in many scientific fields, including surface finish analysis. The concept of fractal dimension is to describe the complexity of a surface in terms of a number. In this paper, the fractal dimension *D_s_* of the three-dimensional topography obtained by scanning before and after interface shearing is also calculated, as shown in Table 6. *D_s_* is distributed between 2.2 and 2.5, and most are approximately 2.3, indicating that the most shearing interfaces are not complex and are closer to flat two-dimensional planes. Under the normal stress, the interface occludes tightly, and the complex bumps, textures, and folds on the interface are all cut off or substantially worn. *D_s_* decreases with the increase of shear times, which is more obvious under the low normal stress. Considering the interface morphology has been seriously degraded after the first shearing under large normal stress, *D_s_* changes little in the subsequent shearing. By contrast, the interface fluctuation gradually degenerates in the process of cyclic shearing due to the shear separation effect between two materials when the normal stress is small. In this case, the reduction of *D_s_* is more distinct.

**Figure 16:**
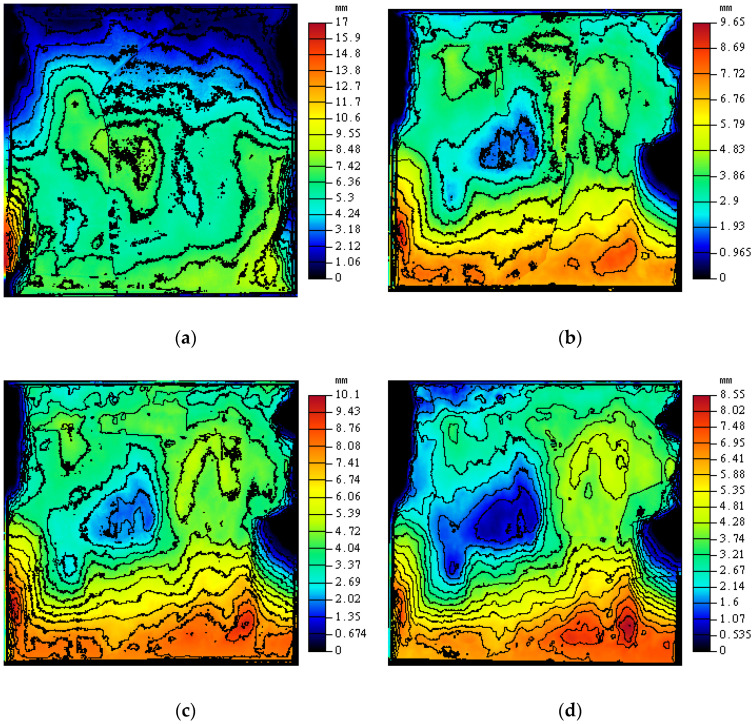
Contour map of CD-3 in cyclic shearing. (**a**) Before test; (**b**) First shearing; (**c**) Second shearing; (**d**) Third shearing.

Figure 16 intuitively shows the morphology changes of the rock–mortar interface in cyclic shearing. With the increase of shear times, the steepness of the interface gradually decreases, and the extreme peak is gradually worn down. In this test, the shear direction is from the top to the bottom. The gentle slope on the surface is mostly toward the shear direction, and the contours along the shear direction become smoother, indicating that the interface convex flattens and the interface sag keeps expanding in the process of cyclic shearing.

**Figure 17:**
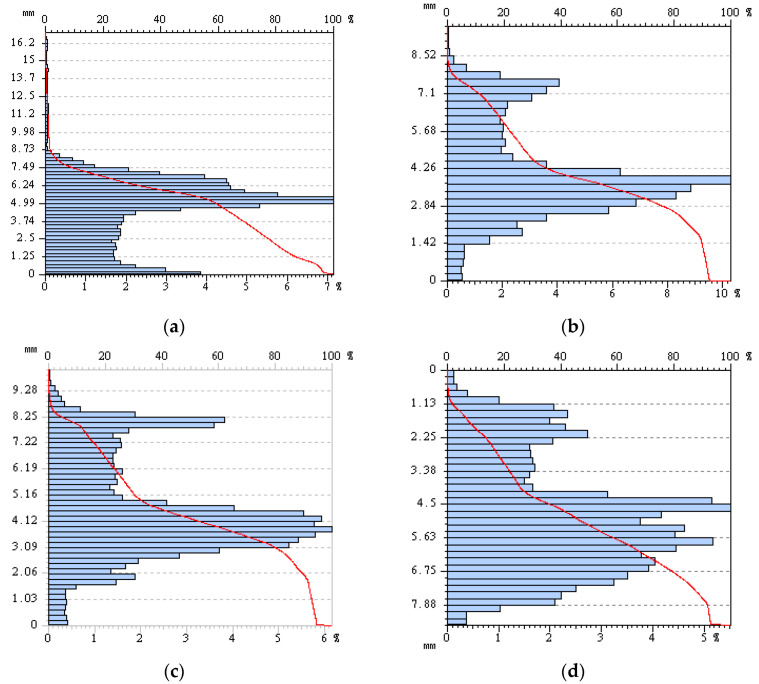
Abbott–Firestone curve of CD-3 in cyclic shearing. (**a**) Before test; (**b**) First shearing; (**c**) Second shearing; (**d**) Third shearing.

The Abbot–Firestone curve shows the statistical distribution of depth at various points on the surface. The vertical axis is graded in height, the lower horizontal axis is graded in percentage of the total number of points, and the upper horizontal axis represents the ratio. The shape of the Abbot–Firestone curve contains information about the finish level on the surface. Figure 17 shows the combination of the Abbot–Firestone curve and height histogram of CD-3 interface. Before casting the mortar, there is a considerable convex on the rock surface, the scale of which could not be judged in the contour map. While in the Abbot–Firestone curve, it accounts for approximately 3%. By comparing the curve shape before and after the test, it is found that the curve fluctuation decreases gradually, indicating that the height distribution on the interface becomes uniform.

### 4.3. Discussions

Shear strength is one of the important properties of interfaces. It is generally believed that interface morphology, normal stress, and the physical and mechanical properties of rock materials are the three main coupled factors that affect shear mechanical behavior of interfaces. Considering that the physical and mechanical properties of rock materials can be measured by laboratory tests and the normal stress can be easily obtained by a variety of monitoring equipment, determining how to characterize the interfaces morphology has become the key issue to be addressed.

The morphology of interfaces plays an important role on the shear and closure properties. Reasonable indexes can fully reflect the geometric features of interface morphology features, such as fluctuation, roughness, and inclination. Therefore, the widely used three-dimensional morphology parameters of rock–mortar interface during cyclic shear tests were calculated to observe the variation trends. In this way, the most sensitive morphology parameters can be selected to associate with the shear strength in physical sense, and a reasonable shear strength prediction model of rock–mortar interface will be obtained. Unfortunately, due to the small number of shearing times, this study failed to establish the relevant prediction model of the cyclic shear strength of rock–mortar interfaces and only presented the changes of each morphology parameter under cyclic shearing effect. In addition, another limitation in this study lies in the fact that the complex lithology of different rock materials is not taken into consideration. Even so, this study can still provide a certain reference for the parameter determination and model construction of subsequent studies by other researchers.

## 5. Conclusions

(1) The failure mode of the rock–mortar interface changes from brittle fracture to interfacial friction in cyclic shearing. The shear strength decreases continuously, and the reduction amplitude decreases gradually. The shear performance of malmstone–mortar interface is better than that of red sandstone–mortar interface, and blue sandstone–mortar interface is the lowest. With the increase of shearing times, the peak dilatancy angle decreases. The greater the normal stress, the greater the drop of peak dilatancy angle. The interface dilatancy angle corresponding to malmstone is larger, followed by red sandstone and blue sandstone.
(2) As the time of shearing increases, both maximum interface height *S_p_* and interface height root mean square *S_q_* decrease. The arithmetic mean difference of surface height *S_a_* also decreases only under the 2 Mpa normal stress, and it fluctuates greatly when the normal stress is 4 Mpa. The variation laws of skew coefficient *S_sk_* and peak coefficient *S_ku_* are not obvious. For more shearing times, the value of the aspect ratio of texture topography ratio *S_tr_* is greater, representing the anisotropy of the rock–mortar interface decreases. Meanwhile, texture direction angle *S_td_* is reduced, especially in the case of high normal stress.
(3) All the surface topography mixed parameters used in this study, including fractal dimension *D_s_*, root mean square of slope of interface *S_dq_*, and interface area ratio *S_dr_*, decrease with the shearing times, wherein when the normal stress is small, the redution of fractal dimension *D_s_* is greater. The increase in shearing times lowers the surface complexity and the the peak curvature, which produces a more rounded peak. Based on the contour map, the interface steepness decreases and the sag continuously expands. According to the Abbot–Firestone curve, it is judged that the height of bearing shear body gradually decreases.

## Figures and Tables

**Table 1:** Mechanics parameters of intact rocks.

Type	UCS/Mpa	Cohesion/Mpa	Friction Angle/°
Malmstone (A)	72.00	9.00	61.00
Red sandstone (B)	29.50	3.93	52.00
Blue sandstone (C)	33.00	2.60	51.70

**Table 2:** The test loading scheme of rock–mortar interface.

Sample Number	Normal Stress/MPa	Shear Rate/mm × s^−1^	Shear Times
AD-1	4	0.01	3
AD-2			
AD-3			
BD-1			
BD-2	2	0.01	3
CD-1			
CD-2			
CD-3			

**Table 3:** The shear strength of different rock–mortar interfaces subjected to cyclic shearing.

Number	First Shearing Stress/MPa	Second Shearing Stress/MPa	Third Shearing Stress/MPa
AD-1	6.08	4.42	4.16
AD-2	6.20	4.35	4.11
AD-3	6.29	4.43	4.32
BD-1	4.88	2.73	2.39
BD-2	3.25	2.15	1.74
CD-1	3.91	1.82	1.61
CD-2	3.08	1.76	1.51
CD-3	2.97	1.73	1.44

**Table 4:** High feature parameters of interface topography before and after shearing.

Interface Number	Topography Parameters	Before Tests	First Shearing	Second Shearing	Third Shearing
AD-1	*S_p_*/mm	2.41	2.36	2.99	2.38
	*S_a_*/mm	1.16	1.19	1.22	1.22
	*S_q_*/mm	1.39	1.5	1.39	1.39
	*S_sk_*	−0.837	−1.45	0.13	0.22
	*S_ku_*	2.89	5.98	1.76	1.75
AD-2	*S_p_*/mm	3.96	4.2	4.07	4.81
	*S_a_*/mm	1.81	2.02	1.62	1.09
	*S_q_*/mm	2.21	2.71	1.85	1.82
	*S_sk_*	−0.386	−1.37	0.31	0.12
	*S_ku_*	3.09	7	1.94	2.12
AD-3	*S_p_*/mm	4.76	4.57	4.23	4.13
	*S_a_*/mm	2.55	1.96	1.81	1.78
	*S_q_*/mm	3.51	2.32	2.12	2.07
	*S_sk_*	−1.25	−0.13	−0.03	0.01
	*S_ku_*	4.11	2.06	2.06	2.01
BD-1	*S_p_*/mm	4.55	4.41	3.06	6.56
	*S_a_*/mm	1.84	2.18	1.61	1.28
	*S_q_*/mm	2.23	2.9	1.51	1.13
	*S_sk_*	−0.72	−1.2	−0.48	0.06
	*S_ku_*	3.13	3.44	2.21	1.62
BD-2	*S_p_*/mm	5.68	4.16	3.69	3.28
	*S_a_*/mm	1.72	1.5	1.43	1.38
	*S_q_*/mm	2.22	1.92	1.89	1.88
	*S_sk_*	−1.41	−0.62	1.14	1.80
	*S_ku_*	4.21	3.14	2.99	1.64
CD-1	*S_p_*/mm	5.27	4.79	4.62	3.7
	*S_a_*/mm	1.47	1.34	1.22	1.18
	*S_q_*/mm	1.82	1.6	1.52	1.4
	*S_sk_*	−0.29	−0.004	0.31	1.38
	*S_ku_*	2.81	2.18	1.97	1.18
CD-2	*S_p_*/mm	5.78	3.28	3.15	2.36
	*S_a_*/mm	2.04	1.11	1.19	0.88
	*S_q_*/mm	2.47	1.34	1.1	0.96
	*S_sk_*	0.01	0.10	0.04	−0.08
	*S_ku_*	2.27	2.23	1.95	1.51
CD-3	*S_p_*/mm	6.88	5.67	5.61	5.06
	*S_a_*/mm	1.75	1.58	1.48	1.24
	*S_q_*/mm	2.14	2.06	2.07	2.03
	*S_sk_*	−0.52	0.08	0.08	0.24
	*S_ku_*	2.71	2.65	2.49	2.33

**Table 5:** Texture characteristics of surface morphology and peak point characteristics evolution.

Interface Number	Topography Parameters	Before Tests	First Shearing	Second Shearing	Third Shearing
AD-1	*S_tr_*	0.383	0.379	0.382	0.384
	*S_td_/°*	48.6	49.6	48.2	48
	*S_pd_*/mm^−2^	0.241	0. 207	0.187	0.176
	*S_pc_*/mm^−1^	0.31	0.291	0.142	0.129
AD-2	*S_tr_*	0.3	0.284	0.309	0.346
	*S_td_/°*	44	46,2	45.7	45.6
	*S_pd_*/mm^−2^	0.314	0.339	0.257	0.252
	*S_pc_*/mm^−1^	0.348	0.677	0.615	0.538
AD-3	*S_tr_*	0.258	0.29	0.371	0.378
	*S_td_/°*	47	46.1	45.9	45.5
	*S_pd_*/mm^−2^	0.782	0.621	0.522	0.494
	*S_pc_*/mm^−1^	0. 802	0.766	0.751	0.732
BD-1	*S_tr_*	0.307	0.311	0.336	0.376
	*S_td_/°*	36.5	36.1	35.7	35.5
	*S_pd_*/mm^−2^	0.491	0.392	0.321	0.301
	*S_pc_*/mm^−1^	0.314	0.302	0.275	0.223
BD-2	*S_tr_*	0.289	0.319	0.325	0.33
	*S_td_/°*	51.2	51	50.7	50.6
	*S_pd_*/mm^−2^	0.411	0.377	0.369	0.362
	*S_pc_*/mm^−1^	0.278	0.241	0.223	0.22
CD-1	*S_tr_*	0.382	0.431	0.436	0.466
	*S_td_/°*	33.3	32	31.5	31.3
	*S_pd_*/mm^−2^	0.583	0.544	0.497	0.437
	*S_pc_*/mm^−1^	0.254	0.196	0.127	0.126
CD-2	*S_tr_*	0.192	0.197	0.263	0.291
	*S_td_/°*	36.1	35.7	35.5	35
	*S_pd_*/mm^−2^	0.732	0.724	0.688	0.685
	*S_pc_*/mm^−1^	0.409	0.351	0.299	0.229
CD-3	*S_tr_*	0.312	0.314	0.32	0.329
	*S_td_/°*	40	36.2	35.6	31.1
	*S_pd_*/mm^−2^	0.781	0.755	0.692	0.663
	*S_pc_*/mm^−1^	0.313	0.282	0.259	0.234

**Table 6:** Mixed feature parameter changes of surface topography.

Interface Number	Topography Parameters	Before Tests	First Shearing	Second Shearing	Third Shearing
AD-1	*S_dq_*	2.13	2.07	1.64	1.3
	*S_dr_*/%	22	30.6	24.17	22
	*D_s_*	2.24	2.43	2.21	2.23
AD-2	*S_dq_*	3.42	3.46	2.11	0.005
	*S_dr_*/%	89.9	109	87.1	86.7
	*D_s_*	2.28	2.29	2.22	2.19
AD-3	*S_dq_*	3.15	3.05	1.72	0.782
	*S_dr_*/%	70.5	84.7	76.5	70.3
	*D_s_*	2.21	2.26	2.29	2.26
BD-1	*S_dq_*	2.48	1.68	1.64	1.62
	*S_dr_*/%	76.8	56.6	49	48
	*D_s_*	2.49	2.29	2.3	2.28
BD-2	*S_dq_*	3.21	2.17	1.88	1.72
	*S_dr_*/%	66	57.3	52.1	50
	*D_s_*	2.31	2.28	2.28	2.21
CD-1	*S_dq_*	3.06	2.57	1.62	1.39
	*S_dr_*/%	51.2	47	42.7	36
	*D_s_*	2.59	2.32	2.23	2.21
CD-2	*S_dq_*	2.749	2.12	0.98	1.297
	*S_dr_*/%	31.9	26.6	16	15
	*D_s_*	2.34	2.29	2.25	2.25
CD-3	*S_dq_*	2.6	2.1	1.71	0.48
	*S_dr_*/%	67	61	54	52
	*D_s_*	2.36	2.3	2.28	2.22

## Data Availability

The data used to support the findings of this study are available from the corresponding author upon request.
